# Beyond Feature Binding: Interference from Episodic Context Binding Creates the Bivalency Effect in Task-Switching

**DOI:** 10.3389/fpsyg.2012.00386

**Published:** 2012-10-05

**Authors:** Beat Meier, Alodie Rey-Mermet

**Affiliations:** ^1^Department of Psychology, Center for Cognition, Learning and Memory, University of BernBern, Switzerland

**Keywords:** cognitive control, binding, anterior cingulate cortex, bivalent stimuli, univalent stimuli

## Abstract

When switching between different tasks and bivalent stimuli occur only occasionally on one of them, performance is slowed on subsequent univalent trials even if they have no overlapping features with the bivalent stimulus. This phenomenon has been labeled the “bivalency effect.” Recent evidence has revealed that this effect is robust, general, and enduring. Moreover, it challenges current theories of task-switching and cognitive control. Here, we review these theories and propose a new, episodic context binding account. According to this account, binding does not only occur between stimuli, responses, and tasks, but also for the more general context in which the stimuli occur. The result of this binding process is a complex representation that includes each of these components. When bivalent stimuli occur, the resulting conflict is associated with the general context, creating a new conflict-loaded representation. The reactivation of this representation causes interference on subsequent trials, that is, the bivalency effect. We evaluate this account in light of the empirical evidence.

Feature binding is essential for the formation of a coherent representation of an object. In addition, binding processes are involved on further levels of information processing and thus, their occurrence is not restricted to the domain of perception. Binding processes are involved in action planning, sensorimotor coordination, and in memory formation (Hommel, 2004; Mather, 2007; Altmann and Gray, 2008; Verguts and Notebaert, 2009) and all these operations are relevant for cognitive control. Cognitive control is necessary in situations in which the course of action must be shielded against distracting events (Botvinick et al., 2001, 2004). For example, when switching between different tasks, which require responding to bivalent stimuli (i.e., stimuli with features that are relevant to more than one task), control is necessary to select the appropriate task and unselect the inappropriate task. In this example, encountering a conflict (i.e., a bivalent stimulus) triggers an adjustment of cognitive control. Here we focus on the adjustment of cognitive control that is induced by the occasional occurrence of bivalent stimuli.

While univalent stimuli trigger one particular task-set, bivalent stimuli trigger two different task-sets and thus can be used to perform two different tasks. In a task-switching environment, examples of univalent stimuli would be black digits presented for a parity decision, black letters presented in uppercase or lowercase for a case decision, or red and blue shapes presented for a color decision. However, when the letters are presented in red and blue color this would turn them into bivalent stimuli. Recent research has demonstrated that when switching among these kinds of tasks even the occasional occurrence of bivalent stimuli results in a general performance slowing that encompasses several subsequent univalent trials. When switching between parity, case, and color decisions with the stimuli introduced in the above example performance is slowed even on those decisions, which shared no relevant feature with the bivalent stimuli (i.e., the parity decisions). This phenomenon has been labeled the “bivalency effect” (Woodward et al., 2003, 2008; Meier et al., 2009; Rey-Mermet and Meier, 2012a,b).

In this article, we provide a review of the empirical findings on the bivalency effect and we show that it challenges established theories of task-switching and cognitive control. So far, the studies on the bivalency effect were driven by the motivation to test alternative explanations. In the course of this work the theoretical notion of “episodic context binding” has emerged as an explanation for the bivalency effect. One goal of the present paper is to relate this account to established theories, set the stage to enable the design of experiments to critically evaluate this new account and to show how it relates to other findings in the literature.

## The Bivalency Effect: An Adjustment of Cognitive Control in Response to Bivalent Stimuli

**Figure 1:**
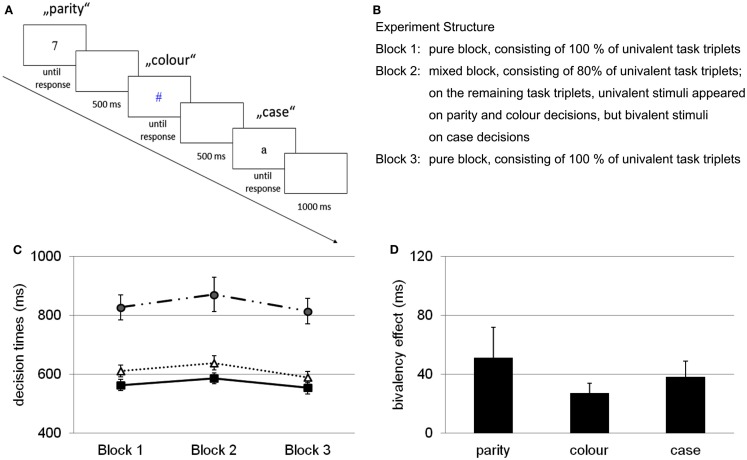
**(A)** Example of the basic paradigm used to test the influence of occasionally occurring bivalent stimuli. A task-triplet comprised a parity decision, a color decision, and a case decision. On a bivalent task-triplet (not pictured here), the letters were presented in color (either in blue or red). **(B)** Experiment structure with three blocks of task-switching; bivalent stimuli occur only in block 2 on 20% of case decisions. **(C)** Decision times for univalent stimuli across blocks (blocks 1 and 3 pure, block 2 mixed), on parity decisions (circles), color decisions (squares), and case decisions (triangles). **(D)** Bivalency effect, expressed as performance difference on univalent stimuli between block 2 and blocks 1 and 3 averaged. Error bars represent standard errors. Adapted from Woodward et al. (2003).

In an initial study, Woodward et al. (2003) used three different binary decision tasks – a parity decision (odd vs. even numerals), a color decision (red vs. blue symbols), and a case decision (uppercase vs. lowercase letters) – and participants were required to repeatedly switch between these tasks, which were always presented in the same fixed order (i.e., parity, color, case).In Figure 1A, an example of the procedure is presented; in Figure 1B the structure of the experiment, consisting of three experimental blocks, is described. In the first and in the last block (i.e., the pure blocks) all tasks involved only univalent stimuli (i.e., black numerals for the parity decision, colored shapes for the color decision, and black letters for the case decision). In the second block (i.e., the mixed block), the stimuli were univalent on most of the trials. However, occasionally, on some of the case decisions (i.e., 20%) the letters were presented in color, thus turning them into bivalent stimuli. With this particular set-up, two tasks included stimuli with overlapping features (i.e., color and case decisions) while one task did not include overlapping stimulus features (i.e., parity decisions). A task-switching paradigm with task-triplets is necessary to test for the effect of occasional bivalent stimuli because a paradigm with only two tasks, which is the standard case in task-switching studies, would always involve an overlap of task features.

The results of this study showed that performance was slowed for bivalent stimuli. However, more critical was the comparison between the performance on the univalent task-triplets of the pure block and performance on univalent task-triplets of the mixed block. These results are presented in Figure 1C. They revealed a performance slowing for all of the tasks from the mixed block, even for the task that involved stimuli that shared no features with the bivalent stimuli. This slowing was coined the “bivalency effect” and is depicted in Figure 1D. Woodward et al. (2003) suggested that these results challenge task-switching theories. These theories have been developed to explain the cost that occurs when switch and repetition trials are compared. They focus primarily on bottom-up processes, that is, processes initiated and guided by the stimuli and their particular features (e.g., Rogers and Monsell, 1995; Allport and Wylie, 2000; Monsell et al., 2000; Meiran, 2008).

For instance, Allport et al. (Allport et al., 1994; Allport and Wylie, 1999, 2000; Wylie and Allport, 2000) proposed a negative priming account. According to this account, when a bivalent stimulus occurs on a given trial, the task-set for the now-relevant task is activated while the task-set for the irrelevant task is inhibited. If the inhibited task becomes relevant on a subsequent trial, additional time is required to reactivate it (i.e., to overcome task-set inertia). Thus, switch costs are the consequence of exogenously triggered processes to resolve interference. Accordingly, a negative priming account can explain the slowing on tasks with univalent stimuli sharing relevant stimulus features with the bivalent stimuli (i.e., the color and case decisions). However, it cannot explain slowing on tasks with univalent stimuli sharing no relevant stimulus features with the bivalent stimuli (i.e., the parity decision).

Similarly, a task-reconfiguration explanation posits that for processing bivalent stimuli an additional decision is required to determine the relevant task-set and switch cost reflects the time needed to reconfigure the task-set (e.g., Fagot, 1994; Rogers and Monsell, 1995; Monsell et al., 2000; Rubinstein et al., 2001; Sohn and Anderson, 2001; Meiran et al., 2008; Braverman and Meiran, 2010). According to this account, univalent stimuli, which share stimulus features with the bivalent stimuli, can activate this additional task-decision process. Specifically, with colored letters for case decisions as bivalent stimuli, the stimuli for the color decision would cue the case decision and an additional process would be required to select the color decision task-set. Similarly, the stimuli for the case decision would cue the color decision and an additional process would be required to select the case decision task-set. However, for univalent stimuli with no overlapping stimulus features, such as those for parity decisions, no additional, time-consuming task-decision process would be required. Thus, this account can explain the slowing on tasks with univalent stimuli sharing relevant stimulus features with the bivalent stimuli. However, it cannot explain the slowing on tasks with univalent stimuli sharing no relevant stimulus features with the bivalent stimuli (i.e., non-overlapping univalent stimuli).

In order to explain the slowing on tasks with non-overlapping univalent stimuli, Woodward et al. (2003) argued that top-down processes are necessary in the sense of a more general adjustment of cognitive control rather than a stimulus-specific effect. Specifically, they suggested that a more cautious response style is triggered by bivalent stimuli. This interpretation was further supported by the finding of a speed-accuracy trade-off, that is, the slowing in Block 2 was also accompanied by an increase in accuracy. However, the latter result was not replicated in the follow-up studies and may have been caused by the particular response requirements of the initial study.

## Testing Explanations for the Bivalency Effect

### Task uncertainty

An alternative interpretation of the initial findings of Woodward et al. (2003) is that rather than an endogenous adoption of a cautious response style, the bivalency effect might represent a process of recovery from task uncertainty elicited by the occasional bivalent stimuli, which would result in a relatively short-lasting effect because only bivalent stimuli induce task uncertainty (Kray and Lindenberger, 2000). To address this possibility, Meier et al. (2009) manipulated the interval between task-triplets and assessed the trajectory of the bivalency effect across task-triplets by presenting bivalent stimuli in the mixed block in regular intervals. They reasoned that the bivalency effect would disappear relatively quickly with longer intervals and across trials with univalent stimuli when it reflects recovery from task uncertainty. In contrast, if the bivalency effect reflects the adoption of a more cautious response style, it should be stable across intervals and should be relatively long-lived.

**Figure 2:**
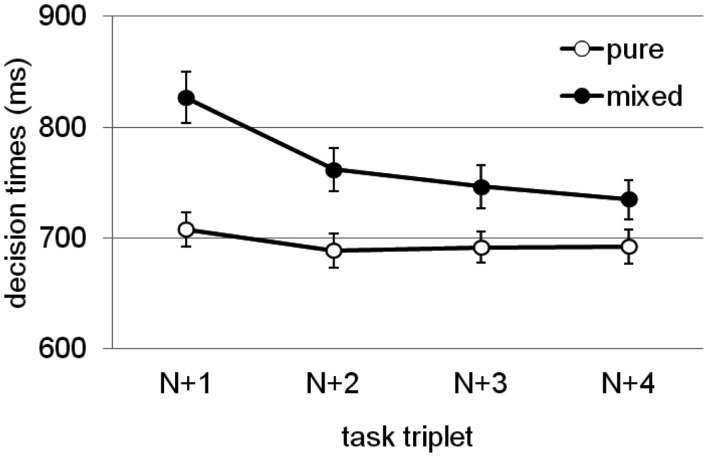
**Endurance of the bivalency effect: Mean decision times for task-triplets following a bivalent case decision in the mixed block (closed circles) compared with the corresponding task-triplets in the pure block**. Error bars represent standard errors. Task-triplet *N* refers to the task-triplet containing a bivalent stimulus in the mixed block; subsequent task-triplets (represented here) are labeled *N* + 1, *N* + 2, *N* + 3, and *N* + 4, respectively. Results adapted from Meier et al. (2009), averaged across experiments, experimental conditions, and tasks.

In three separate experiments with a similar set-up as Woodward et al. (2003), but with variations of the specific tasks, modalities, and bivalent stimuli, Meier et al. (2009) found a consistent bivalency effect across all experiments and experimental conditions. Further, the bivalency effect was not reduced by increasing the interval between task-triplets, and it was still present four task-triplets after the occurrence of a bivalent stimulus. The trajectory of the bivalency effect across task-triplets, averaged across experiments, experimental conditions, and tasks, is illustrated in Figure 2. It shows that although there is a steady decline in its size, the bivalency effect is characterized by a long-lived slowing. In the condition with the longest inter-trial interval, responding on a task-triplet took on average approximately 8 s (required for making three decisions, each requiring approximately 600 ms, plus two 500 ms inter-stimulus-intervals, plus the 5000 ms interval). Thus, the occasional occurrence of a bivalent stimulus was sufficient to slow down decision making on univalent stimuli for at least half a minute. Meier et al. reasoned that such a long-lasting effect cannot solely be attributed to temporary task uncertainty. Figure 2 shows that the decline is steepest from first trial following a bivalent stimulus to the second subsequent trial. This may indicate that the bivalency effect involves two separate components. One that is short-lived and related to task uncertainty, or potentially to an orienting response to an infrequent event (cf. Notebaert et al., 2009; Nùñez Castellar et al., 2010; Notebaert and Verguts, 2011), and another one that is long-lived and rather related to a persisting adjustment of cognitive control such as the adoption of a more cautious response style.

Meier et al. (2009) noted that the episodic binding of tasks, stimuli, and the experience of trickiness (i.e., episodic context binding) may have contributed to the bivalency effect. They reasoned that a stimulus acquires a history during an experiment, that is, it acquires an association with the context in which it occurs (see Waszak et al., 2003; Hommel, 2004; for similar notions). If episodic binding is not only specific to stimuli and tasks, but also extends to the context in which they occur (i.e., among purely univalent stimuli or among univalent stimuli and occasionally occurring bivalent stimuli), univalent stimuli and tasks are bound to the more demanding context created by bivalent stimuli. Episodic binding would occur whenever a series of events is (co-)registered such as when performing a task-triplet in the present task-switching experiments. According to this “episodic context binding” explanation, conflict is bound to the context in which bivalent stimuli have been encountered (i.e., a triplet of tasks) and on subsequent univalent trials, this representation is reactivated and slows performance on all of the trials, even on those with no overlapping features.

### Response-set priming

Another alternative possibility for the occurrence of the bivalency effect is related to the fact that the same responses have been used for each of the three tasks, that is, due to overlapping response-set. According to this explanation, rather than episodic context binding or endogenous adaptation of a cautious response style, the conflict produced by the bivalent stimulus may be bound to the particular response. Because each of the three tasks in a task-triplet shares the same response-set, the conflict associated with a particular key-press in response to a bivalent stimulus can slow down performance when this particular key-press is required on subsequent univalent trials. According to this explanation, the bivalency effect would result from negative priming of bivalent stimulus features via shared response features. This hypothesis is fueled by theoretical and empirical considerations of priming from response features to stimuli (e.g., Deubel and Schneider, 1996; Paprotta et al., 1999; Hommel et al., 2001; Kunde and Kiesel, 2006; Fagioli et al., 2007; Metzker and Dreisbach, 2009).

**Figure 3:**
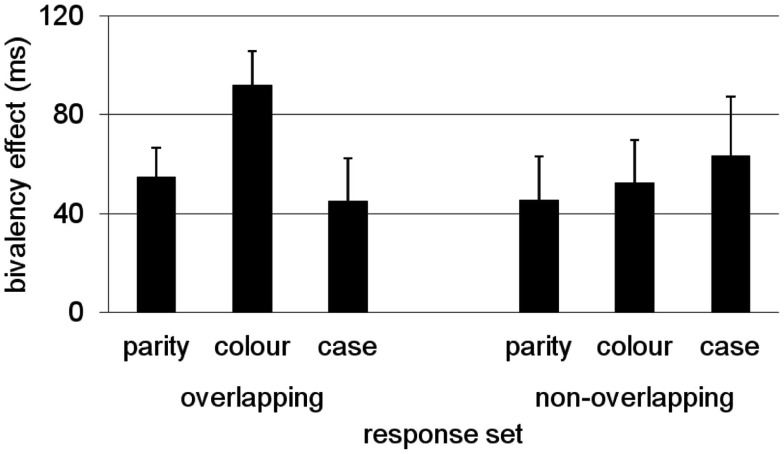
**Invariant bivalency effect across response-set conditions depicted as DT difference between univalent stimuli from the mixed block and the average of the pure blocks**. Results adapted from Rey-Mermet and Meier (2012a) averaged across experiments.

To test this hypothesis, Rey-Mermet and Meier (2012a) conducted a study in which they contrasted a condition with an overlapping response-set (as in previous studies) and a condition in which responding to each task was mapped on two separate effectors (non-overlapping response-set). They reasoned that if bivalent stimuli prime conflict via response features, then using a non-overlapping response-set would reduce conflict priming, particularly for those tasks that do not share the same responses. In contrast, if the manipulation of response-set does not affect the pattern and magnitude of the bivalency effect, this would rather suggest that the bivalency effect is due to episodic context binding. In two separate experiments, in which the order of tasks was varied, the results showed a consistent bivalency effect that was not affected by the type of response-set (i.e., overlapping vs. non-overlapping). These results, that is, the bivalency effect across response-set conditions, averaged across experiments, are presented in Figure 3. It is important to note that despite some non-significant variability between tasks and conditions, a significant bivalency effect was present even for parity decisions in the non-overlapping response-set-condition. Thus, the bivalency effect cannot simply be due to response-set priming.

Rey-Mermet and Meier (2012a) related the findings to results from Waszak and Pholulamdeth (2009) who observed that an emotionally arousing picture modulated the episodic binding between a stimulus and a task. They interpreted these results as support for an episodic context binding explanation and suggested that a context does not even need to be emotionally arousing to have an impact on performance. Rather it is sufficient when it triggers a conflict, such as the trickier context caused by occasionally occurring bivalent stimuli (cf. Verguts and Notebaert, 2009, for similar considerations).

### Conflict specificity

In the basic paradigm that was used to investigate the bivalency effect, only task-switching trials were present (Figure 1A). As switch trials require the inhibition of the previously relevant task and the activation of the newly relevant task, they inherently involve a conflict (Rogers and Monsell, 1995; Allport and Wylie, 2000). Moreover, for some of the trials, a second source of conflict was present due to feature overlap between univalent and bivalent stimuli (cf. Allport and Wylie, 2000; Waszak et al., 2003; Meiran, 2008). So far, the results indicate that the magnitude of the bivalency effect is not dependent on the amount of conflict, that is, from one source such as a switch trial, or from two sources such as a switch trial with features that overlap with the bivalent stimulus. However, it is not clear whether the bivalency effect would occur in the absence of any conflict. To test this question, Rey-Mermet and Meier (2012b) introduced repetition trials into the basic paradigm. Thus, participants were required to perform six rather than only three decisions. Specifically, they were asked to perform repeatedly a series of two size decisions (large vs. small), two parity decisions (even vs. odd), and two letter decisions (vowel vs. consonant), conforming to an AABBCC-scheme (where ABC refer to the three different tasks). Across three experiments, the order of the tasks – but not the scheme – was varied. Moreover, in Experiment 1, bivalent stimuli were created by presenting some of the letters for the consonant-vowel decisions either in large or small font; in the other two experiments bivalent stimuli were created by presenting some of the digits either in large or small font for the parity decisions (Experiment 2) or for the size decisions (Experiment 3).

The question whether the bivalency effect would have a differential impact on switch and repetition trials is also important for the interpretation of switch costs (i.e., the slower performance on switch compared to repetition trials). As noted above, one interpretation of switch costs is that they reflect control processes that reconfigure the cognitive system in order to switch tasks (e.g., Rogers and Monsell, 1995; Meiran, 1996). Another interpretation is that they arise from the negative priming of stimulus and task features (e.g., Allport and Wylie, 2000; Waszak et al., 2003). Both interpretations are concerned with what switch costs represent, making it important to understand which factors affect them in task-switching procedures. Moreover, if the bivalency effect contributes to switch costs, it would reflect a so far neglected component of switch costs.

**Figure 4:**
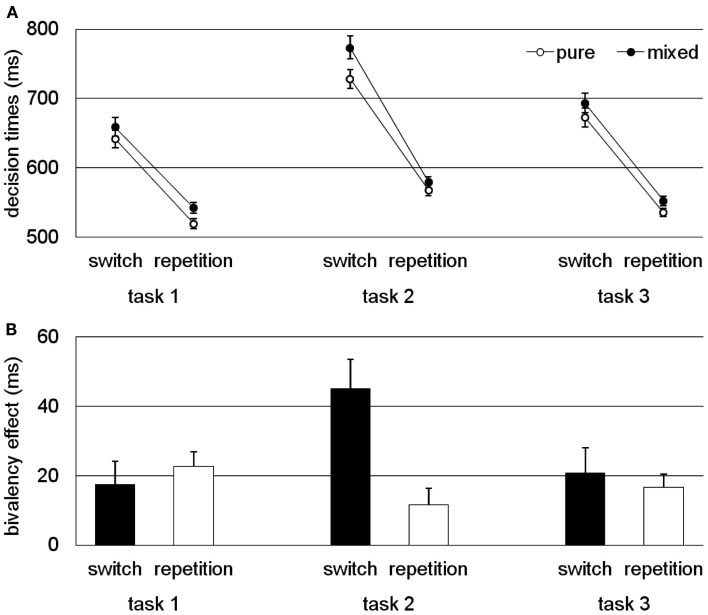
**Conflict specificity of the bivalency effect (task 1 refers to the task with overlapping stimulus features, task 2 refers to the task with no overlapping stimulus features, and task 3 refers to the task that occasionally involved bivalent stimuli)**. **(A)** Decision time data (i.e., performance on univalent stimuli for switch and repetition trials in pure and mixed blocks). **(B)** Bivalency effect (i.e., difference between univalent trials from the pure block and those from the mixed block). Results adapted from Rey-Mermet and Meier (2012b) averaged across experiments and tasks.

The results are summarized in Figure 4. In Figure 4A, the decision times for pure and mixed blocks are presented, in Figure 4B the bivalency effect is presented. Overall, the results showed a consistent bivalency effect for all the conditions in which at least one source of conflict was present. However, it was largely reduced and statistically not significant in two of the three experiments for the condition with no conflict, that is, the repetition trials for the parity decision in Experiment 1 and the letter decisions in Experiment 3. Switch costs were affected only for the particular task with no overlapping stimulus features. Thus, for typical task-switching studies that involve two tasks and stimuli with overlapping features by design, the bivalency effect is leveled out by calculating switch costs as the difference between DTs on switch and repetition trials.

Rey-Mermet and Meier (2012b) suggested that the bivalency effect reflects a flexible adjustment of cognitive control, which is sensitive to the presence of conflict, but neither to its amount nor to its source. The occasional occurrence of bivalent stimuli induces an adjustment of control that is sufficient to deal with situations with an additional source of conflict at no cost. However, it seems to be sensitive to the mere presence of conflict and thus the need for resource allocation is reduced for non-conflict trails (i.e., task repetitions with non-overlapping stimulus features).

The results challenge a prominent hypothesis in cognitive control research, namely that the adjustment of cognitive control is always sensitive to the amount and to the source of conflict (e.g., Botvinick et al., 2001, 2004; Egner, 2008). They also indicate that the bivalency effect does not stem from a general adoption of a more cautious response style. According to this explanation, the presence or absence of conflict on a particular decision should not have affected the magnitude of the bivalency effect. In contrast, an episodic context binding account would suggest that interference is only invoked when a conflict-loaded representation of a task is reactivated. Specifically, the degree of the association between a particular task and its context (i.e., the strength of binding) seems to depend on the presence of conflict (cf. Verguts and Notebaert, 2009). Accordingly, the relationship between presence of conflict and binding is responsible for the reduction of the bivalency for repetition trials.

## The Neural Basis of the Bivalency Effect

**Figure 5:**
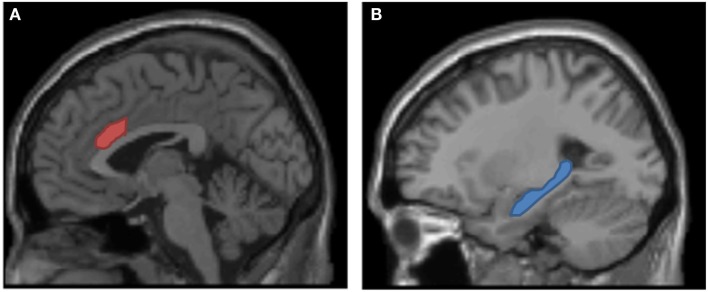
**Neural structures underlying the bivalency effect**. **(A)** Dorsal anterior cingulate cortex signals the requirement to adjust cognitive control (cf. Woodward et al., 2008). **(B)** Hippocampus (and other memory-related structures not depicted here) are required for episodic binding and the reactivation of the episodic context (cf. Meier et al., submitted).

From a neuropsychological view, adjustment of cognitive control in response to conflict is typically associated with increased activation in the dorsal anterior cingulate cortex (dACC, Figure 5A). The functions of this brain area include conflict detection, modulation of conflict, and selection between competing mental processes and task-sets (Botvinick et al., 1999; Peterson et al., 1999; Cohen et al., 2000; Holroyd and Coles, 2002; Forstmann et al., 2006; Parris et al., 2007). As the dACC is involved in situations in which an adjustment of the course of action is necessary to overcome obstacles and to meet the actual goals, one would expect that it is also involved in the bivalency effect. To test this expectation, using an event-related functional resonance imaging (fMRI) design, Woodward et al. (2008) contrasted univalent stimuli from a condition with purely univalent stimuli and univalent stimuli from a condition in which bivalent stimuli were occasionally intermixed on one of the tasks (cf. Figure 1). As expected, the results showed that the bivalency effect was associated with activation in the dACC. Similarly, using event-related potentials, Grundy et al. (2011), found amplitude differences at frontal electrodes within time windows of 275–450 and 500–550 ms. They interpreted these modulations as “suppression of processing carried over from irrelevant cues.” Moreover, consistent with the fMRI results, source dipole analyses revealed dipole locations at or close to the dACC.

Thus, there is converging evidence that the bivalency effect is associated with activations in brain areas that signal conflict processing or adjustment of cognitive control. However, it is not clear, what exactly triggers conflict in the absence of bivalent stimuli, that is, when processing purely univalent stimuli. According to the “episodic context binding account” the reactivation of a representation of conflict that has been built up by processing the conflict-loaded task-triplet is a likely explanation. If we consider that binding processes take place on each trial (i.e., stimuli, tasks, and task-triplets acquire a history, cf. Waszak et al., 2003; Meier et al., 2009) then we would also predict memory-related brain activations. However, when contrasting blocks with bivalent stimuli vs. blocks without bivalent stimuli in an fMRI or ERP-study these activations cancel each other out. Thus, the results from neuroimaging and electrophysiological studies do not contradict the “episodic context binding account.”

However, from these considerations it is clear that we would also expect that memory-related brain areas are necessary for the occurrence of the bivalency effect (in particular the hippocampus, cf. Figure 5B). One possibility to investigate this expectation is to test amnesic patients. Amnesic patients have a profound deficit in memory binding, in particular binding an event to a particular context (e.g., Chun and Phelps, 1999; Hannula et al., 2006; Pascalis et al., 2009). Thus, if episodic context binding is involved in the bivalency effect, amnesic patients would be expected to show a considerable reduction in the magnitude of the bivalency effect. In a recent study involving severely memory-impaired amnesic patients, this hypothesis was confirmed (Meier et al., submitted). Although the patients were able to perform the task and they were slowed when processing bivalent stimuli, they did not show a bivalency effect. This result supports the notion that memory-related brain areas are involved in the reactivation of the conflict and that both the hippocampus and the dACC are neural foundations of the bivalency effect.

## Episodic Context Binding as Source of Interference

The definition of “context” may be at the core of the relation between the proposed “episodic context binding account” and other previous notions of episodic binding (e.g., Waszak et al., 2003; Hommel, 2004; Altmann and Gray, 2008; Verguts and Notebaert, 2009). Given that the basic paradigm used to establish the bivalency effect involves a regular sequence of decision tasks, context is established through the repeated sequential presentation of task-triplets. From the point of view of a participant, a task consists of the whole sequence of the three different decision tasks (e.g., parity – color – case) rather than being composed of three separate tasks. Thus, the representation of a particular decision task includes the context of the whole task-triplet. When a bivalent stimulus is presented on one of these tasks, the conflict that is triggered spreads to the representation of the whole context. For the next task-triplet, this representation is reactivated and performance is slowed for all the stimuli, even for those that have no overlapping features with the bivalent stimulus. If one considers “context” identical to “task-set,” the current approach would be quite similar to other binding theories of cognitive control. In fact, for example Hommel (2004) acknowledged that “binding takes place across domains, linking relevant, or salient features to the response it is accompanied by and the task-set it is processed in” (p. 498).

To answer the question how exactly the context is established further research is necessary. For example, it would be interesting to address whether the sequential presentation of the tasks is necessary or whether the bivalency effect would occur in a task-cuing context. By switching between task-triplets that involve and triplets that do not involve stimuli that are related to the task-triplets with the bivalent stimuli the specificity of the context binding can be further tested. Moreover, it will be important to see whether the findings generalize to other paradigms such as the Simon- or the Flanker-tasks.

Recent research has suggested that two qualitatively distinct control modes operate to fine tune cognitive control processes, retroactive control, and proactive control (Braver et al., 2007; Braver, 2012). In fact, research on the bivalency effect has been concerned with retroactive control, that is, where resources are recruited in a just-in-time manner when conflict is detected. Defining bivalent stimuli by instructions (e.g., as in prospective memory research) will allow the investigation of conflict anticipation and particularly, to test whether this would produce a similar adjustment of control (cf., Meier and Rey-Mermet, 2012).

A further issue is the definition of binding. It is important to note that the terms “binding” and “association” are closely related. It has been suggested that “binding” rather indicates a “momentary or short-lived coupling of elements in the service of a task,” while association refers to a “long-term coupling of elements” (Vandierendonck et al., 2010, p. 607). However, according to Vandierendonck et al. (2010) binding can also be considered as a “short-term association that may be kept in long-term memory if the elements involved in the binding are not needed in other coupling that could interfere with the already existing association” (Vandierendonck et al., 2010, p. 607). To be consistent we have referred to the critical process as “episodic context binding” rather than to “episodic context association” in our work.

## Episodic Context Binding in a Broader Context

So far, we have focused on the episodic context binding account with respect to the bivalency effect. In this final section we will highlight that it is also related to other findings in the domain of task-switching and cognitive control. Specifically, an episodic context binding account can contribute to the explanation of phenomena such as switch costs, mixing costs, and *N*−2 repetition costs.

### Switch cost

The bivalency effect may be present in most task-switching studies because these studies typically involve bivalent stimuli throughout. However, as already noted by Woodward et al. (2003), the confounding of switch cost proper and the bivalency effect is probably minimal in task-switching paradigms that involve only two tasks because switch and repetition trials typically occur within the same block. Similarly, the bivalency effect would also exert a comparable cost when switching between and repeating of multiple tasks is involved, as long as all of these tasks involve bivalent stimuli only. Thus, the bivalency effect would exert a relatively equal influence on both switch and repetition trials and would be canceled out when switch costs are computed (see also Rey-Mermet and Meier, 2012b).

Moreover, the episodic context binding account is also compatible with results from task-switching studies in which task-switching performance on univalent stimuli was compared with task-switching performance on univalent stimuli that appeared intermixed with bivalent stimuli. In the seminal study by Rogers and Monsell (1995), in which the AABBAABB-design was introduced into the literature, they found slower responses to stimuli that appeared intermixed with bivalent stimuli compared to a condition in which task-switching was carried out with univalent stimuli only (Rogers and Monsell, 1995, Experiment 1). However, they did not further discuss this finding. From an episodic context binding view, the performance slowing on univalent stimuli in the intermixed trials can be easily explained by the notion that the typical context, in which the particular task occurred, involved conflict and this conflict was reactivated even on those trials that did not involve bivalent stimuli.

### Mixing costs

Mixing costs refer to the difference between repetition trials in mixed blocks (consisting of both switch and repetition trials) and single task blocks (i.e., with repetition trials that are univalent by definition) with the typical finding of slower performance on mixed blocks compared to single task blocks. Mixing costs have been considered as a confound in early task-switching studies in which single task repetition blocks and alternating task blocks have been compared to measure switch costs (e.g., Jersild, 1927; Spector and Biederman, 1976). Specifically, the task-switch variable is confounded by working memory demands, attentional requirements, and degree of arousal (Rogers and Monsell, 1995; Meiran, 1996). However, mixing cost can be considered as an important indicator of executive control (Braver et al., 2003; Rubin and Meiran, 2005). For example, Kray and Lindenberger (2000) found that mixing cost was strongly affected by old age, while switch cost was not.

Slower responding has been observed under task repetition conditions when a series of tasks contained regular switch trials (such as in the AABBAABB-design), but also when a series of tasks contained only a few switch trials compared to pure task repetition conditions (De Jong, 2000, Exp. 2; Mayr, 2001; Kray et al., 2002; Exp. 2). De Jong (2000) interpreted these results as the consequence of a control strategy that may reflect a compromise between minimizing control effort and maximizing task performance. Specifically, participants may opt not to fully disengage prior task-sets when they have the expectation that they may become relevant again on subsequent trials. In contrast, according to an episodic context binding account this adjustment may reflect rather the result of memory processes, that is, the association or binding between a task and a conflict-loaded context which is reactivated even in a context that is not conflict-loaded.

### *N*−2 repetition costs

*N*−2 repetition costs refer to the performance difference between *N*−2 task switches (i.e., a sequence such as CBA) and *N*−2 task repetitions (i.e., sequences such as ABA). Interestingly performance is slowed for an *N*−2 repetition (ABA) compared a non-repetition control condition (CBA). This slowing has been interpreted as a measure of inhibitory processes in the selection of task-sets (Mayr and Keele, 2000; Gade and Koch, 2007, 2012). It is assumed that after having performed task A, task-set A is inhibited in order to successfully perform task B. When encountering task A again, inhibition is still active and overcoming task A inhibition in order to perform task A again requires time, which is reflected in the *N*−2 repetition cost. Typically, the *N*−2 cost is tested with tasks that involve bivalent stimuli. However, in order to test whether the size of the *N*−2 repetition cost is related to the amount of conflict among tasks, a recent study has included some trivalent stimuli (i.e., 25%) amongst the bivalent stimuli (Gade and Koch, 2012). The critical question was whether the presence of a univalent vs. trivalent stimulus on trial *n*−1 would affect performance on *N*−2. The results showed no effect of stimulus valence and thus, Gade and Koch suggested that inhibitory processes are engaged in a rather global manner, which is consistent with an episodic context binding account. Even more interestingly, an additional result was that performance on the intermixed univalent stimuli did not differ from the corresponding bivalent stimuli. Because there was no change in context in which the particular task had been activated previously, this result is exactly what would have been predicted by an episodic context binding account.

Overall, these results show that the episodic context binding account can be used to explain several findings that have occurred as side-effects in the study of switch costs, mixing costs, and *N*−2 costs. Thus, the occurrence of episodic context binding is not restricted to the bivalency effect and the episodic context account complements and extends existing theories.

## Conclusion

In this article, we have reviewed the emerging literature on the bivalency effect. The bivalency effect refers to the phenomenon that the occasional occurrence of bivalent amongst univalent stimuli slows performance on subsequent univalent trials, even on those, that share no relevant feature with the bivalent stimulus. From these studies it is evident that this effect challenges current theoretical approaches of task-switching and cognitive control.

Specifically, the slowing observed on stimuli, which share no relevant features with the bivalent stimuli, cannot be accounted for by task-switching theories. However, to be fair it must be noted that these theories have been developed to explain switch costs in the first place. Accordingly, task-switching theories can predict the slowing on those univalent stimuli that have shared properties with the bivalent stimuli. In contrast, the episodic context binding account can explain the slowing on each type of stimulus in terms of binding and reactivation of conflict and context. Thus, it is beyond feature binding. Rather it is related to episodic memory such as establishing an association between tasks and contexts.

However, episodic context binding is engaged flexibly, depending on the presence or absence of conflict (Rey-Mermet and Meier, 2012b). These results challenge the hypothesis that adjustment of cognitive control is always sensitive to the amount and to the source of conflict (e.g., Botvinick et al., 2001, 2004; Egner, 2008). Rather they indicate that the presence of a conflict in univalent trials strengthens binding whereas the absence of conflict weakens it (cf. Verguts and Notebaert, 2009). In summary, considering the general context in which a task occurs informs both theories of task-switching and cognitive control.

## Conflict of Interest Statement

The authors declare that the research was conducted in the absence of any commercial or financial relationships that could be construed as a potential conflict of interest.
